# Web-Based Interventions to Promote Healthy Lifestyles for Older Adults: Scoping Review

**DOI:** 10.2196/37315

**Published:** 2022-08-23

**Authors:** Audrey Lavoie, Véronique Dubé

**Affiliations:** 1 Faculty of Nursing Université de Montréal Montreal, QC Canada; 2 Université de Montréal Marguerite-d’Youville Research Chair on Humanistic Nursing Interventions Montreal, QC Canada; 3 Research center Centre Hospitalier de l'Université de Montréal Montreal, QC Canada

**Keywords:** aged, behavior change, components, effects, healthy lifestyle, web-based intervention

## Abstract

**Background:**

With the aging of the population and rising rates of chronic diseases, web-based interventions could be considered to support older adults in adopting healthy lifestyles. To date, published knowledge syntheses have focused on quantitative studies among older adults aged ≥50 years. However, those aged ≥65 years may have different needs to be met by these interventions because of the biological and physiological changes associated with aging, and qualitative studies could help advance knowledge in this field.

**Objective:**

The objective of this scoping review is to explore the extent of the literature on web-based interventions aimed at promoting healthy lifestyles among people aged ≥65 years.

**Methods:**

A scoping review was conducted based on the framework proposed by Levac et al. Six databases (ie, MEDLINE, CINAHL, PsycINFO, Web of Science, the Cochrane Database of Systematic Reviews, and the Joanna Briggs Library) and gray literature (ie, Google Scholar and OpenGrey) were searched. The final search was conducted on June 23, 2021. The studies were selected by 2 persons (AL and ML) independently. The included studies were systematic reviews and qualitative and quantitative studies focusing on web-based interventions to promote healthy lifestyles in people aged ≥65 years that were published in French or English between 1990 and 2021. Data were extracted in a table and synthesized based on the conceptualization of web-based interventions (ie, according to the use parameters, behavior change techniques, delivery modes, and theories). A thematic analysis was performed.

**Results:**

In total, 20 articles were included in this review, which represents studies focused on 11 distinct interventions. All of the interventions (11/11, 100%) aimed to promote physical activity among older adults. The number of intervention sessions varied from 5 to 16, with a frequency from daily to once every 2 weeks. Diverse delivery modes such as electronic diary, video, and phone call were found. The most used behavior change techniques were instruction, feedback, and self-monitoring. Few interventions (6/11, 55%) were based on a theory. A favorable trend was observed in increasing physical activity, and 5 themes emerged that appeared to be central to behavior change among older adults: motivation, support, tailoring, barriers, and perceptions.

**Conclusions:**

This scoping review provides a better understanding of the components of web-based interventions and their outcomes on the healthy lifestyles of people aged ≥65 years. These findings could provide important guidance for the design and development of future web-based interventions in this field. Further research is needed to continue the development and evaluation of innovative and accessible interventions to promote healthy lifestyles among older adults.

**International Registered Report Identifier (IRRID):**

RR2-10.2196/23207

## Introduction

### Background

The number of older adults worldwide (ie, those aged ≥65 years) is expected to almost double over the next 30 years, from 12% to 22% [1]. This significant aging of the population will not be without consequences for health care systems. In fact, this phenomenon will lead to an increase in the rate of chronic diseases considering that the prevalence of these diseases increases with age and that older adults are seriously affected by them [2,3]. In this regard, healthy lifestyle habits (ie, good nutrition, regular physical activity [PA], smoking abstinence, the limiting alcohol consumption, and the management of stress) could help prevent a significant number of diseases in older adults [2], promote longevity [4], reduce frailty [5], and maintain health [3]. For these reasons, older adults should benefit from interventions that support their adoption of healthy lifestyles.

Moreover, with the advances in health technologies, the web is increasingly the preferred method of intervention even among older adults, whose internet use has been growing rapidly in recent years [6-8]. Web-based interventions can be defined as care or treatments that aim to promote behavior change and that are delivered via a web browser over the internet on different technological tools such as computers, tablets, or cell phones [9]. Web-based interventions can take many forms, such as educational programs, disease management programs, and web-based group exercise programs; can include different technologies such as artificial intelligence algorithms or monitoring devices; and can be self-guided or human-assisted [10]. Web-based interventions could be used to support individuals as they adopt healthy lifestyles and would be favorable for older adults [11,12]. In addition, such interventions constitute an economical and accessible alternative for health care systems [13]. In the context of a global pandemic, lifestyle habits such as sedentary behavior and dietary changes may also be disrupted [14], and older adults’ access to programs and services to facilitate the adoption of healthy lifestyles, such as gyms, can be limited [15]. As a result, web-based interventions may represent a solution for helping older adults adopt and maintain a healthy lifestyle [15].

In addition, the current global pandemic makes web-based interventions all the more relevant as the modes of intervention delivery need to be reconsidered to promote social distancing [16,17]. The recommendations on social distancing must be followed to preserve the population’s health, especially among vulnerable older adults. However, despite the current need for social distancing, we need to ensure that this mode of intervention delivery is as suitable as in-person interventions [16]. In particular, as older adults place a high value on trusting relationships in behavior change, human contact needs to be preserved through any web-based interventions that are introduced to support their adoption of healthy lifestyles [18,19]. Among other things, human contact could be maintained by including the support of a coach, which would also increase the commitment of older adults to the intervention [20]. Although the literature documents numerous web-based interventions, their components and effects are diverse, making it difficult to draw any conclusions about which components promote optimal change outcomes in this population [21]. In this regard, Webb et al [22] developed a framework to facilitate investigations of the components of web-based interventions that will optimally influence behavior change. They found that web-based interventions that incorporate behavior change techniques (BCTs) and that are theoretically grounded lead to better outcomes in terms of health behavior change and that delivery modes could also affect such change [22].

Although some authors have published syntheses of the literature focused on web-based interventions designed to promote lifestyle changes in adults [11,23], to our knowledge, no synthesis of knowledge has focused on people aged ≥65 years. In fact, there appears to have been only 2 systematic reviews conducted on web-based interventions focused on healthy lifestyle habits for people aged ≥50 years [24,25]. The primary studies included in these systematic reviews had small sample sizes with an average age of ≥50 years, which means that they did not focus specifically on a population of older adults [24,25]. There is another review of the literature that examined web-based interventions to promote PA in older adults. This review included primary studies with samples of older adults aged ≥55 years and other age groups as well (ie, adults) [26], which means that the interventions included were not specific to older adults. Although there is no consensus in the literature on the specific age used to define old age, the World Health Organization [2] suggests defining older adults as persons aged ≥65 years. Frequently, it is assumed that interventions designated for young or middle-aged people will be adapted for use with older adults [27]. However, older adults are a heterogeneous group with multiple characteristics, and those aged ≥65 years may have different needs to be met by the interventions because of the biological and psychological changes associated with aging, such as decreased functional capacity, frailty, and changes in social position [2,28]. Moreover, older adults should be able to benefit from accessible health services that are adapted to their needs [29], and any interventions developed for them must consider the challenges associated with aging [27]. In this sense, as the components of the interventions as well as their outcomes could differ for adults aged ≥65 years, it is essential to explore the literature dealing with this specific population.

The 3 syntheses of the literature that we found on web-based interventions among people aged ≥50 or ≥55 years focused only on quantitative studies [24-26]. However, the literature also provides qualitative studies on this subject, and they can make relevant contributions to the components and outcomes of web-based interventions designed to promote healthy lifestyles among older adults. For example, qualitative studies may provide more information about the experiences of older adults who participate in web-based interventions, particularly with regard to the components of the intervention, which is important for the development of knowledge in this area and for future studies. A scoping review on this topic would appear appropriate as it will permit an exploration of the available literature by including both qualitative and quantitative studies. To our knowledge, no study has explored the extent of knowledge of web-based interventions for people aged ≥65 years by including both qualitative and quantitative studies.

### Objectives

Therefore, the purpose of this study is to explore the extent of the literature on web-based interventions aimed at promoting healthy lifestyles among people aged ≥65 years.

## Methods

### Overview

A scoping review was conducted based on the framework proposed by Levac et al [30]. According to Levac et al [30], a scoping review may be conducted to determine the scope of the research or map the available literature on a phenomenon, which is the purpose of this review. This review followed the 5 steps of the framework developed by Levac et al [30], as presented in the following sections. The protocol for this scoping review is available elsewhere [31].

### Identifying the Research Questions

The research questions were identified following a brief review of the initial literature and discussions with the research team, which was composed of a doctoral student (AL), a researcher (VD), and a librarian (RB). This scoping review will seek to answer the following questions: (1) What are the web-based interventions aimed at promoting healthy lifestyles among people aged ≥65 years? (2) What are the components of these interventions (ie, use parameters, BCTs, delivery modes, and theories used)? (3) What are the reported outcomes of these interventions?

### Identifying Relevant Studies

To identify relevant studies, the following databases were consulted: MEDLINE, CINAHL, PsycINFO, Web of Science, the Cochrane Database of Systematic Reviews, and the Joanna Briggs Library. These databases were selected for their focus on the social and health sciences, the field related to the topic of this study. Gray literature was searched using the Google Scholar and OpenGrey databases. The reference lists of the identified articles were checked to ensure that all the relevant articles had been included. The authors of the primary studies were contacted when additional information was required.

The search strategy used keywords and descriptors related to the concepts of older adults, lifestyle, and web-based interventions. The complete search strategies for each database are presented in Multimedia Appendix 1. The criteria for inclusion were (1) articles published between 1990 and 2021 as the World Wide Web was created in 1989 [32]; (2) articles published in French or English; (3) articles related to the objective of the scoping review, that is, a web-based intervention delivered via a web browser over the internet addressed to a population of older adults and aimed at promoting healthy lifestyle habits (ie, diet, regular PA, smoking abstention, limiting the alcohol consumption, and management of stress); and (4) primary studies such as experimental studies, quasi-experimental and qualitative studies, systematic reviews, and other documents associated with gray literature (such as government reports and clinical practice guidelines). Research protocols were also included as they often provide a more in-depth description of the intervention studied. For the purpose of this study, web-based interventions were defined as care or treatments aimed at changing behavior and accessed via a web browser over the internet [9]. The scope excluded teleconsultations with health care professionals and websites that provided information without any interaction. Articles were excluded when (1) persons aged ≥65 years were not the population specifically studied; (2) the web component of the intervention was not predominant, such as a face-to-face intervention that was complemented by a web-based component; and (3) healthy lifestyle habits were not primarily targeted by the intervention, such as symptom self-management programs that included some physical exercise. Finally, the identified articles were exported to a data management software program (ie, Covidence) where duplicates were removed. The final database search was performed on June 23, 2021.

### Study Selection

The studies were selected by 2 independent persons (AL and ML). An initial selection was made by reading the abstracts and titles of the articles, and then the selected articles were read in full, retaining only those related to the purpose of the study and the research questions and that met the established inclusion and exclusion criteria. In cases where there was disagreement over a selection, a third person (VD) was consulted. As suggested by Levac et al [30], the 2 persons who selected the studies met at the beginning, midpoint, and end of the selection process to clarify any difficulties they had encountered and revise the research strategy. Inclusion and exclusion criteria were clarified in terms of the definition of the web-based interventions (ie, delivered via a web browser over the internet regardless of the technological tool used, such as a tablet or a computer) to be included as well as the population (ie, excluding studies that targeted multiple age groups) and the behavior targeted (ie, excluding web-based interventions for fall prevention that included some exercises). To promote transparency, a PRISMA (Preferred Reporting Items for Systematic Reviews and Meta-Analyses) diagram was used to illustrate the study selection process and present the excluded articles and the reasons for their exclusion.

### Charting the Data

Data were extracted into a table including the authors, year and location of publication, purpose, type of study, population and sample, method, intervention and comparison, intervention components, and outcomes. As suggested by Levac et al [30], data from the first 5 papers were extracted independently by 2 persons (AL and ML) to ensure compliance. As the purpose of the review was to explore the breadth of knowledge rather than assess the rigor of the studies identified, the quality of the studies was not assessed.

### Collating, Summarizing, and Reporting the Results

To collect, synthesize, and report the results, we used a conceptualization of web-based intervention components proposed by Webb et al [22]. As mentioned previously, this conceptualization seeks to classify the components of web-based interventions into 3 categories: the BCTs used, the delivery modes, and the theories used. Webb et al [22] conceived this framework based on the BCT taxonomy developed by Michie et al [21], on a coding scheme for classifying delivery modes, and on the coding theory scheme by Michie and Prestwich [33]. In its most recent version, the taxonomy by Michie et al [21] details 93 BCTs as strategies used in interventions to promote behavior change, including feedback, action planning, and instruction, among others. The coding scheme for the delivery modes includes additional modes for the web component. It can be used to categorize them as automated functions (eg, video, automated tailored feedback, and automated following messages such as reminders or encouragement), communicative functions (eg, chat session, peer-to-peer access, and “ask the expert facility”) and additional modes (eg, email, phone calls, and videoconferencing) [22]. The Michie and Prestwich [33] coding scheme contains questions to assess whether and how theories are used in an intervention. Synthesizing the components of the web-based interventions into these categories (ie, BCTs, delivery modes, and theories) facilitates an understanding of the components that can have an influence on health behavior change. We also used additional items in the CONSORT-EHEALTH (Consolidated Standards of Reporting Trials of Electronic and Mobile Health Applications and Online Telehealth) guidelines proposed by Eysenbach [9] for reporting eHealth trials. This guideline proposes including use parameters such as the number of sessions, duration of the intervention, and frequency when reporting web-based intervention components.

### Analysis

To summarize the data by examining the components and outcomes of the interventions studied, we drew on the method of thematic analysis used by Paillé and Mucchielli [34] to analyze the data. More specifically, components of the web-based interventions were grouped according to their similarity, divergence, complementarity, or recurrence. Themes were identified from the extracted data based on the results of the interventions. Again, these themes were grouped into thematic clusters (ie, groups of themes with common characteristics). For example, the themes “tailored content to each participant” and “personalized advice in regard to preference and condition” could be grouped together into the thematic cluster “tailoring.” The analysis was carried out by the first author (AL) and then validated by the second author (VD). The PRISMA-ScR (Preferred Reporting Items for Systematic Reviews and Meta-Analyses extension for Scoping Reviews) checklist [35] was used to ensure that all the key items were reported and promote study replicability (Multimedia Appendix 2). A narrative summary is presented in the next section as an overview of the components and outcomes of the interventions studied.

## Results

### Search Results

**Figure 1:**
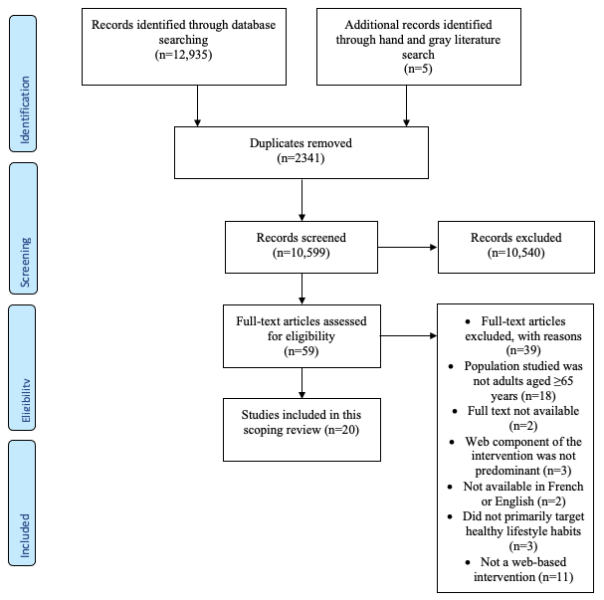
PRISMA (Preferred Reporting Items for Systematic Reviews and Meta-Analyses) flow diagram of the literature search and article selection process.

Initially, 12,940 articles were identified. After removing duplicates, 10,599 articles were filtered, and 10,540 (99.44%) of these were excluded by reading the article titles and abstracts based on the inclusion and exclusion criteria. Finally, 0.56% (59/10,599) of the articles were read in their entirety. A total of 20 articles were included in this review. The main reason for excluding an article was when it had a study population that was not specifically older adults aged ≥65 years. The PRISMA diagram shown in Figure 1 summarizes the process used to identify and select the studies.

### What Are the Web-Based Interventions Aimed at Promoting Healthy Lifestyles Among People Aged ≥65 Years?

Among the 20 articles included, we found studies focused on 11 distinct interventions: Active for Life (n=1, 5%), Active Plus (n=2, 10%), Active Plus 65 (n=2, 10%), eMIND (n=2, 10%), Healthy Ageing Supported by Internet and Community (n=1, 5%), Health Aging Through Internet Counseling in the Elderly (n=6, 30%), Life Project (n=1, 5%), MyPlan 2.0 (n=1, 5%), and Otago (n=2, 10%), plus 2 other interventions not named [36,37]. The range of publication years was 2013 to 2021, and most of the studies (19/20, 95%) were published in the last 5 years. These studies were mainly published in the Netherlands (9/20, 45%), France (3/20, 15%), Australia (1/20, 5%), Italy (2/20, 10%), Switzerland (1/20, 5%), Spain (1/20, 5%), Belgium (1/20, 5%), Finland (1/20, 5%), and the United States (1/20, 5%). In total, of the 20 studies, there were 6 (30%) randomized controlled trials, 4 (20%) research protocols, 5 (25%) qualitative studies, 3 (15%) pretest-posttest studies, 1 (5%) randomized pilot study, and 1 (5%) mixed methods study. The sample sizes identified varied from 16 to 2624 participants. The interventions targeted older adults aged ≥65 years living in the community [38] who could walk without technical help (2/20, 10%) [39-41], were prefrail (1/20, 5%) [42], were inactive (3/20, 15%) [36,37,43], had a chronic disease with a disability (2/20, 10%) [44-47], presented 2 or more cardiovascular risk factors or an antecedent of cardiovascular disease (1/20, 5%) [12,19,20,48-50], or were at risk of cognitive decline (1/20, 5%) [51,52]. The average age of the study samples ranged from 68.7 to 83 years, with an average age of 73 years, which means that the studies targeted younger older adults. Indeed, only 20% (4/20) of the studies (3/11, 27% of the interventions) had a sample with an average age of ≥75 years [36,42,46,47].

Most of the articles (9/20, 45%) were focused on evaluating the effects of a web-based intervention on the health behaviors of older adults, including PA and diet, or on other variables, including cardiovascular risk factors such as diabetes, obesity, hypertension, and hypercholesterolemia; self-efficacy [12]; knowledge and skills [38]; and cognitive function [12,51]. Other articles presented a description of the intervention (6/20, 30%) or the experience of the older adults with regard to their participation in the intervention (5/20, 25%), such as the appreciated component, reason for participation, or preferences. All the interventions (11/11, 100%) targeted PA, and 73% (8/11) of them targeted PA as the only behavior. In total, 18% (2/11) of the interventions targeted PA in addition to other behaviors such as nutrition [51,52] and nutrition and alcohol consumption [38]. A total of 9% (1/11) of the interventions targeted all the cardiovascular risk factors such as PA, blood pressure, diabetes, weight, nutrition, and smoking cessation, and participants could choose their health priorities [12,19,20,48-50]. A summary table of the study characteristics is available in Multimedia Appendix 3 [12,19,20,36-52]. The components of the interventions (ie, use parameters, BCTs, delivery modes, and theories) are presented in the following sections.

### What Are the Components of These Interventions?

#### Use Parameters

Use parameters refer to the duration of the intervention, the duration of each session, the number of sessions, and their frequency. In this review, the *duration of the intervention* varied from 5 weeks to 18 months. Only 18% (2/11) of the interventions reported the *duration of each session* [37,39,40]. It would have been helpful to know the duration of each session in the other studies to understand how much time older adults need to spend to complete the sessions. The *number of sessions* varied from 5 (1/11, 9%) [41] to 16 (1/11, 9%) [39,40]. Although the total number of sessions was not specified, in some interventions, the authors proposed an intensity of intervention such as 5-minute daily sessions (1/11, 9%) [37] or free access during the entire study period (2/11, 18%) [36,51,52]. Otherwise, in 45% (5/11) of the interventions, the number of sessions was not reported [12,19,20,38,42,44-50], which does not allow us to understand how many web-based intervention sessions older adults require. The *session frequency* was daily [37], twice per week [39,40], weekly [41], and once every 2 weeks [43]. In 45% (5/11) of the interventions, the frequency of sessions was not reported [12,19,20,38,42,44-50]. These were the same studies that did not report the number of sessions.

#### BCTs Component

BCTs are the strategies used in interventions to promote behavior change, such as feedback, action planning, or instruction [21]. On the basis of the taxonomy by Michie et al [21] comprising 93 BCTs, we identified 15 different BCTs used in the web-based interventions. The number of BCTs used varied from 1 to 9, with an average of 4.5, meaning that all the interventions except 1 (10/11, 91%) [42] combined multiple BCTs. As the combination of BCTs was varied, it is difficult to establish links between the most used or effective combinations and the results obtained and discern the contribution made by each of them. However, we identified a trend in the combination of instruction, self-monitoring, and feedback (7/11, 64%) despite the fact that this combination was mostly coupled with one or more other BCTs such as action planning or goal setting.

All the interventions (11/11, 100%) used *instructions* on how to perform the behavior. Most of the interventions included *feedback* on the targeted health behavior (9/11, 82%) [12,19,20,36,37,39-41,43-45,48-50] and *self-monitoring* of the behavior (8/11, 73%) [12,19,20,36-41,43,48-52]. Other BCTs used included *action planning* (5/11, 45%) [36,41,43-47], *goal setting* (4/11, 36%) [12,19,20,37,41,43,48-50], *problem solving* (4/11, 36%) [12,19,20,37,41,46-50], *awareness* (1/11, 9%) [46,47], *verbal persuasion* (1/11, 9%) [39,40], *commitment* (1/11, 9%), *self-regulation* (1/11, 9%) [44,45], *prompts and cues* (1/11, 9%), *rewards* (1/11, 9%), *social comparison* (1/11, 9%), and *relapse prevention* (1/11, 9%) [43]. Although common techniques were identified, they were rarely explained by the authors, which makes their definition and application unclear. In fact, only 18% (2/11) of the interventions provided detailed discussions on how the BCTs were used to generate the desired change [41,43]. For example, Alley et al [43] detailed that action planning was used by asking questions about the participants’ actions in terms of what, when, and where to perform the behavior. Several other BCTs from the taxonomy by Michie et al [21], such as distraction, self-affirmation, or scheduled consequences, were not identified in this review. For a summary of the BCTs used in each web-based intervention, see Multimedia Appendix 4 [12,19,20,36-52].

#### Delivery Modes

The delivery modes include every mode in addition to the web component grouped as follows: automated function (eg, video, automated tailored feedback, and automated following messages such as reminders or encouragement), communicative function (eg, chat session, peer-to-peer access, and “ask the expert facility”), and additional modes (eg, email, phone calls, and videoconferencing).

##### Automated Function

All interventions (11/11, 100%) included behavioral information such as how to stay active, guidelines on PA, a workout plan, and safety when exercising. Some interventions included videos on how to modify behavior (4/11, 36%) [12,19,20,36,38,42,48,49], an electronic diary to track behavior (2/11, 18%) [12,19,20,36,38,42,48,49], a quiz (1/11, 9%) [41], and reminders to use the platform either sent by email (2/11, 18%) [41,43] or provided throughout the platform (1/11, 9%) [12,19,20,48-50]. Many interventions (5/11, 45%) offered automated, tailored feedback based on individual progress either throughout the platform [37,41,43] or by email [44-47]. Among these, in the case of 9% (1/11) of the interventions, a combination of automated and personal feedback was provided [12,19,20,48-50].

##### Communicative Functions

A total of 27% (3/11) of the interventions included a messaging system that offered the possibility of chats with a coach [12,19,20,39,40,48,49,51,52], and 18% (2/11) proposed a chat forum with peers [38-40]. In 9% (1/11) of the interventions, participants received written feedback from a physiotherapist [36], but it remains unclear whether this was automated or personal. Some interventions (2/11, 18%) also included in-person meetings, such as an initial meeting with the coach [12,19,20,36,48,49] and a monthly peer mentor meeting [36]. Other interventions included the possibility of training with peers on the web (1/11, 9%) [39,40] and receiving phone calls from a member of the research team (1/11, 9%) [36]. Some interventions (4/11, 36%) also offered participants an opportunity to take part in local group activities [12,19,20,42,44-49], but the authors provided no information on how many participants took part in these activities.

##### Supplementary Modes

The studies included in this scoping review were focused on a web-based intervention but, as discussed previously, some of them also included a supplementary delivery mode such as email (3/11, 27%) [41,44-47], phone calls (2/11, 18%) [10,17,18,36,48-50], and face-to-face contact (2/11, 18%) [12,19,20,36,48,49]. No intervention used SMS text messaging or videoconferencing. However, as most of the studies that included supplementary modes (7/8, 88%) did not provide information on the impacts of these additional modes on behavior change in older adults, it is difficult to know how such modes influenced the results.

#### Theory

The coding scheme developed by Michie and Prestwich [33] allows for an assessment of the extent to which the interventions are theory-based. In total, 55% (6/11) of the interventions were based on at least one theory. A total of 18% (2/11) of the interventions were based on 1 theory, whereas 36% (4/11) were based on 2 to 5 theories. The theories used were the theory of planned behavior (2/11, 18%), social cognitive theory (2/11, 18%), precaution adoption process (1/11, 9%), integrated model for change (1/11, 9%), self-regulation theory (3/11, 27%), transtheoretical model (2/11, 18%), self-determination theory (2/11, 18%), motivational interviewing (1/11, 9%), I-Change Model (1/11, 9%), and health action process approach (1/11, 9%).

Among the theory-based interventions, most of the studies (5/6, 83%) did not explicitly state how the theory was used to develop the intervention or how it was integrated into the intervention to lead to the desired change. Therefore, it is difficult to understand the contribution made by the theories used and link them to the results obtained. This was despite the fact that the authors of the papers on all the theory-based interventions specified that they wanted to act on constructs of the theory related to the targeted behavior change, such as self-efficacy, motivation, or attitudes, yet they did not provide definitions of the constructs or explanations of how they operationalized them. Definitions and explanations of the constructs would be needed to understand how the intervention attempted to act on them and lead to behavior change [53]. In only 18% (2/11) of the interventions [41,43] did the authors link theory constructs with the BCTs used. For example, Alley et al [43] indicated that the BCTs instruction and feedback were used to change the attitudes of the participants. In other studies (9/11, 82%), no connection was made between the BCTs and theory constructs when this would have helped us understand how the chosen BCTs would lead to the desired change in terms of the constructs targeted. Otherwise, the authors of the papers on 18% (2/11) of the interventions measured a construct of the theory, such as self-efficacy, as an outcome of the study [12,43]. The authors of the papers on 9% (1/11) of the interventions specified that the theory’s constructs were used to tailor the intervention to the participants such that the content of the advice depended on the intrinsic motivation of the participant [44,45]. Table 1 presents a summary of the components of the interventions surveyed.

**Table 1:** Summary of the intervention components (N=11).

Intervention	Population and behavior	Use parameters	Delivery mode	Behavior change technique	Theory
Active for Life [43]	Older adults aged ≥65 years who did not meet the recommendations for PA^a^ PA	Duration of the intervention: 12 weeks Duration of each session: NR^b^ Number of sessions: 6 Frequency: bimonthly	Automated function: tailored feedback on PA via the platform based on the participants’ characteristics Communicative function: none Additional modes: none	Instruction Goal setting Self-monitoring Action planning Prompts and cues Rewards and relapse prevention Social comparison Feedback	Theory of planned behavior Social cognitive theory
Active Plus [44,45]	Older adults aged ≥65 years who had at least one chronic disease that affects mobility and were able to walk 100 m without help PA	Duration of the intervention: 4 months Duration of each session: NR Number of sessions: NR Frequency: NR	Automated function: tailored advice on PA and feedback via email Communicative function: none Additional modes: list of local group activities	Instruction Action planning Coping planning Commitment Self-regulation Feedback	Theory of planned behavior Precaution adoption process Integrated model for change Self-regulation model
Active Plus 65 [46,47]	Older adults aged ≥65 years with an impairment in PA caused by a noncommunicable chronic disease PA	Duration of the intervention: 4 months Duration of each session: NR Number of sessions: NR Frequency: NR	Automated function: tailored advice on PA via email Communicative function: none Additional modes: list of local group activities and email	Instruction Problem solving Action planning Coping planning Awareness Feedback	I-Change Model Transtheoretical model Self-determination theory Self-regulation theory Health action process approach
eMind [51,52]	Community-dwelling older adults aged ≥65 years who presented a subjective memory complaint without dementia PA and nutrition	Duration of the intervention: 6 months Duration of each session: NR Number of sessions: NR Frequency: free access	Automated function: tailored exercise program, nontailored nutritional advice, and website link to a cognitive training Communicative function: chat with health professionals anytime and chat with a dietician for people at risk of nutritional deficiency Additional modes: none	Instruction Feedback Self-monitoring with activity tracker	NR
Healthy Ageing Supported by Internet and Community [38]	Older adults aged ≥65 years PA, nutrition, alcohol consumption, and social participation	Duration of the intervention: 10 weeks Duration of each session: NR Number of sessions: NR Frequency: NR	Automated function: information on physical (food and drink), social (preventing loneliness), and emotional (eg, self-esteem and resilience) health and videos Communicative function: chat forum Additional modes: none	Instruction and self-monitoring	NR
HATICE^c^ [12,19,20,48-50]	Older adults aged ≥65 years with high cardiovascular risk Smoking, blood pressure, cholesterol, diabetes, weight, PA, and nutrition	Duration of the intervention: 18 months Duration of each session: NR Number of sessions: NR Frequency: NR	Automated function: tailored lifestyle and cardiovascular feedback, electronic diary, educational content, and peer-to-peer videos Communicative function: personal and automated feedback from a coach with the possibility to chat Additional modes: 12-month phone call and list of local group activities	Instruction Goal setting Self-monitoring Problem solving Automated and personal feedback	Motivational interviewing Transtheoretical model Social cognitive theory
Life Project [42]	Prefrail older adults aged 74 to 91 years PA	Duration of the intervention: NR Duration of each session: NR Number of sessions: NR Frequency: NR	Automated function: healthy lifestyle and PA information, exercise videos, and the possibility to create a tailored program Communicative function: none Additional modes: list of local group activities	Instruction	Self-determination theory
MyPlan 2.0 [41]	Older adults aged 65 to 80 years able to walk 100 m without help PA	Duration of the intervention: 5 weeks Duration of each session: NR Number of sessions: 5 Frequency: each week and free access	Automated function: information about PA, quiz about PA and benefits, and tailored feedback Communicative function: none Additional modes: email reminders	Instruction Computer-tailored feedback Goal setting Problem solving Action planning Self-monitoring	Self-regulation theory
Otago [39,40]	Community-dwelling older adults aged ≥65 years who were not frail PA	Duration of the intervention: 8 weeks Duration of each session: 30 to 40 minutes Number of sessions: 16 Frequency: 2 times per week	Automated function: exercise instruction and plan Communicative function: possibility to communicate with a coach and peers Additional modes: possibility to train with peers on the web	Instruction Feedback Self-monitoring Verbal persuasion	NR
No name [37]	Inactive older adults aged ≥65 years PA	Duration of the intervention: 2 months Duration of each session: 5 minutes Number of sessions: NR Frequency: every day	Automated function: exercise instruction and examples through an embodied conversational agent Communicative function: none Additional modes: implementation of the intervention in a clinic waiting room for 12 months	Instruction Self-monitoring Feedback Goal setting Problem solving	NR
No name [36]	Older adults aged ≥70 years with self-reported impaired balance, able to rise from a high chair and stand without support, and not active PA	Duration of the intervention: NR Duration of each session: NR Number of sessions: NR Frequency: NR	Automated function: tailored PA information, exercise video, and diary Communicative function: feedback from a physiotherapist, peer mentor meeting once a month, phone calls from a researcher after 2 to 3 weeks, and optional phone support Additional modes: first meeting in group, phone call, and face-to-face meeting	Instruction Action planning Self-monitoring Feedback from a therapist	NR

^a^PA: physical activity.

^b^NR: not reported.

^c^HATICE: Healthy Ageing Through Internet Counselling in the Elderly.

### What Are the Reported Outcomes of These Interventions?

#### Outcomes

Of the 6 studies that evaluated the effects of web-based interventions on PA in older adults, 4 (67%) found positive outcomes on PA after the intervention, including increasing weekly minutes of moderate to vigorous PA and greater likelihood of performing self-reported cycling [37,41,45,46]. Other positive effects were also reported on blood pressure, lipid levels, BMI, smoking cessation, self-efficacy [12], and participants’ knowledge and skills to adopt a healthy lifestyle [38]. Among the 35% (7/20) of studies that conducted a qualitative evaluation, 5 themes emerged from the thematic analysis, which are detailed in the following sections: tailoring, motivation, support, barriers, and perceptions.

#### Theme 1: Tailoring

Several studies (4/7, 57%) addressed the concept of tailored web-based interventions. Indeed, older adults mentioned that they appreciated participating in a web-based intervention tailored to their limitations and preferences [20,42]. Some participants mentioned that the exercises proposed in the web-based intervention were too easy and repetitive, not adapted to their environment [51], or of limited value owing to their medical condition [36]. This suggests the need for web-based interventions tailored to older adults’ preferences, environments, and conditions.

#### Theme 2: Motivation

Motivation appeared to be central to behavior change among older adults as most studies (6/7, 86%) addressed it. Participants stated that web-based interventions should help increase their motivation for change [19,51]. Identifying their own motivation is important for older adults to maintain behavior change [36], and the sources of such motivation varied, including personal benefits and health improvement [19,36]. Some participants mentioned that the coach’s positive message could help boost their motivation [20,51] and that being motivated helped them continue using the intervention and vice versa [20]. Other participants argued that behavior change, such as being more physically active, was not a goal in itself but rather that they were motivated by other health benefits such as remaining independent as long as possible [42]. For these reasons, it would appear necessary to explore the individual motivations of each older adult to facilitate change.

#### Theme 3: Support

The theme of support was reported in several studies (5/7, 71%). Older adults mentioned that they need support to achieve their health goals [19] and that the platform could provide continuous support [20], especially as training alone at home requires discipline [42]. Some participants mentioned that they appreciated having discussions with a coach throughout the web-based intervention [19,40]. Some participants also argued that a first meeting with the coach was necessary to develop a relationship of trust and then facilitate change and that the coach played an important role in stimulating the initial use of the web-based intervention and sustaining it [20]. In other words, participants who feel connected with the coach are more likely to keep using the platform and continue pursuing goals for lifestyle changes [20]. In addition, participants in the study by de Souto Barreto et al [51] would have appreciated having more contact with a member of the research team, suggesting that, as older adults, they would have been favorable to having the support of a coach. Conversely, peer interactions were less valued and used by older adults [40].

#### Theme 4: Barriers

Some barriers were consistently identified in the studies (4/7, 57%) on the use of web-based interventions among older adults. First, older adults mentioned barriers to the web-based interventions, such as a lack of computer skills [19,20] or using an old computer [42]. Difficulties encountered in computer use or limited internet skills could discourage older adults from using a web-based intervention [20]. Some participants also mentioned that they sometimes lacked the discipline required to exercise alone at home [36,42]. Without the support of a coach, older adults feared getting hurt [42], which could be a barrier to behavior change.

#### Theme 5: Perceptions

Behavior change among older adults seemed to be influenced by their perceptions of their age and the benefits of change. Indeed, older adults who do not perceive a need to improve their lifestyles or who do not prioritize it because of their advanced age are less likely to use a web-based intervention and, therefore, engage in lifestyle changes [20]. By contrast, perceiving that behavior change could lead to health benefits positively influences older adults toward using the intervention [19,20]. However, this theme emerged in only 29% (2/7) of the studies, which is why it may have had less impact than the other themes.

## Discussion

### Principal Findings

#### Overview

This scoping review sought to explore the extent of the available literature on web-based interventions as a way to promote healthy lifestyles among people aged ≥65 years. In total, 11 different interventions discussed in 20 published articles were included in this review. Almost all the articles (19/20, 95%) were published in the last 5 years, which indicates growth in the development and evaluation of this type of intervention among older people. This is consistent with the increased use of the web by older adults in recent years [6-8] and the urgent need to deploy cost-effective strategies to facilitate access to health care [13-15].

As found in other studies [22,54], our results show that the studies included predominantly young older adults, with few that took an interest in the “oldest old” such as persons aged ≥85 years. As the literature is so limited on web-based interventions involving people of more advanced age (eg, ≥85 years) and as the components and effects of web-based interventions may differ for this population, further studies are needed across the aging spectrum. This scoping review found that web-based interventions among older adults are mainly focused on increasing PA. This high prevalence could be explained by the fact that older adults are considered the most sedentary age group [55] and that the benefits of PA are considerable for this population [56]. Given that other lifestyle habits such as diet, stress, and alcohol consumption [2] would also be favorable to the health of older adults, more studies should be conducted to evaluate the effects of web-based interventions on these habits in this population.

#### Components

The web-based interventions included in this review had various components. The interventions were diverse in terms of their use parameters (ie, duration, number of sessions, completion time, and frequency). In almost all the interventions (10/11, 91%), at least one detail regarding the use parameters was omitted, making it difficult to understand the intensity of the interventions offered to older adults. As noted in the studies examining preferences toward web-based interventions, older adults prefer a few 30-minute sessions [57] or shorter 10-minute sessions on a regular basis every 2 or 3 days [57,58]. Among adults, other studies have shown that web-based interventions that are more intensive [24], that allow for longer durations, such as 60-minute sessions or more, and that propose a total number of sessions of >3 [59] are more effective at producing behavior change [24,59]. In this review, because of a lack of detailed information on use parameters, it was difficult to identify any trend in use parameters that were more relevant to supporting change among older adults. Further research is needed to better investigate the optimal use parameters (ie, duration, number of sessions, completion time, and frequency) of web-based interventions for older adults.

In this review, we found that the most used BCTs were instruction, feedback, and self-monitoring, which is similar to the findings of other studies that explored the use of BCTs in web-based interventions [22,60]. However, BCTs were used in combination without explaining why these choices were made and how they were operationalized. For this reason, it was difficult to discern the contribution of each BCT to the results obtained [61] and how they could have led to change [53]. BCTs are the active ingredients in an intervention to effect behavior change. Therefore, it is crucial for authors to be explicit about their choice of BCT combinations to understand how interventions produce their effects [62]. Although some interventions (2/11, 18%) observed that some BCTs such as self-regulation could be effective for adults and not for older adults [63,64], further research is needed to understand which BCTs are more appropriate to support older adults in their adoption of healthy lifestyles. In particular, further studies are needed to explore which combinations of BCTs could optimize the intervention’s impact and how each BCT interacts with the others within an intervention to produce behavior change among older adults [62].

The results of this review show the diverse range of delivery modes used in web-based interventions. Some included an electronic diary (2/11, 18%), quiz (1/11, 9%), or videos (4/11, 36%) as well as supplementary modes such as phone calls (2/11, 18%), face-to-face meetings (2/11, 18%), and email reminders (3/11, 27%). All the interventions (11/11, 100%) provided instructions on how to perform the behavior, which other authors have pointed out as the core of most web-based interventions [65]. Many (5/11, 45%) proposed automated feedback, which would be one of the most effective delivery modes leading to behavior change in adults [26]. Only 9% (1/11) of the interventions included a forum with peers, and it was underused by participants, which is inconsistent with other studies in which adults aged ≥50 years showed high use [66] and appreciation [67]. Few interventions (3/11, 27%) offered a chat with a coach, which is similar to the findings of other studies on web-based interventions among adults [22,59]. In the interventions that did offer a chat with a coach, participants were offered an opportunity to communicate with a coach if needed rather than for constant support. The actual nature, dose, and type of coaching provided by this coach was poorly reported by the studies. However, as pointed out by other authors, the constant support of a coach throughout a web-based intervention could replace the sense of interpersonal connectedness found in in-person interventions [68], which older adults seek [18,69]. A systematic review also found that web-based interventions that include human support are more effective at behavior change among middle-aged and older adults than stand-alone interventions [24]. That being said, although a certain delivery mode could be more appropriate for older adults [27], the literature on this subject is limited. More studies, such as meta-analyses, are needed to identify which delivery modes are more effective at inducing behavior change among older adults. This could guide the design of future interventions. Further studies should also investigate web-based interventions that give older adults the choice to participate in a group forum, as well as different forms of coaching by a professional throughout the web-based interventions. More studies are needed to examine the nature of the role played by the coach throughout a web-based intervention as well as explore the dosing and type of support needed to help older adults adopt healthy lifestyles. For us, it seems clear that this coaching could be provided by a health professional such as a nurse as it is a nurse’s role to support people in health promotion and older adults appreciate developing a trusting relationship with a nurse [69].

In this review, few of the interventions (6/11, 55%) were based on a theory. The most common theory reported was the self-regulation theory. This differs from other reviews, in which one mainly finds the social cognitive theory in behavior change interventions among middle-aged [70] and older adults [71]. Among the theory-based interventions, most of the studies (5/6, 83%) did not report how the theory was used to design an intervention that would lead to the desired change. For this reason, it is difficult to draw conclusions regarding the theories and the results obtained. Theories help explain why and how behavior change occurs and provide guidance on the potential determinants to be targeted by the intervention to induce behavior change [62]. In addition, designing interventions based on theories allows us to link the theoretical determinants of behavior change with intervention components [33], know which BCTs to use [62], and ensure that the intervention will lead to behavior change. Indeed, it is well known that theory-based interventions are more effective than non–theory-based interventions [72], and this has also been demonstrated in a population of older adults [71]. Future web-based interventions to promote healthy lifestyles among older adults should be based on theory, and researchers should clearly state how theories guide the development of their interventions. Further studies are needed to compare interventions based on different theories in terms of the effects identified on the lifestyles of older adults.

#### Outcomes

As reported in previous studies [65,70], we observed a favorable trend in the use of web-based interventions to increase PA among older adults. This review found that web-based interventions can also have positive effects on blood pressure, lipid levels, BMI, smoking cessation, self-efficacy and knowledge, and the skills needed to adopt a healthy lifestyle. This is consistent with research findings on the benefits of web-based interventions [24,25].

As a result of our analysis, 5 themes emerged that appear to be central to web-based lifestyle change interventions among older adults: tailoring, motivation, support, barriers, and perceptions. As has been pointed out by many authors [29,73-75], the results of this review show that motivation is one of the most important factors influencing the lifestyle habits of older adults. As motivation is an intrinsic factor (ie, each person must identify their own), increasing individual motivation among older adults may facilitate behavior change [76]. In this sense, future web-based interventions among older adults should target this determinant as a way to help them adopt healthy lifestyle habits [26]. This review also found that older adults appear to appreciate interventions that include support from a coach, which also supports their motivation for change and engagement with the intervention. These findings are consistent with other studies in which older adults mentioned that support and the development of a relationship of trust are necessary in behavior change interventions [18,29,77]. This finding may inform the development of future web-based interventions intended to promote healthy lifestyles among older adults by including the support of a coach.

In line with the results of other studies [57,78], this review highlighted the fact that older adults would prefer interventions that are tailored to their preferences and conditions. Indeed, it appears that tailored web-based interventions can make older adults more engaged in behavior change [79] and lead to better recall of information [80]. Previous studies have suggested that tailored web-based interventions are more effective at inducing behavior change than generic interventions in a middle-aged adult population [25,26]. For older adults, designing a tailored web-based intervention appears to be even more important considering the heterogeneity of this population and the various challenges associated with aging, including comorbidities and frailty, which are experienced differently by older adults [27]. Consistent with the findings of other authors [57,79], this review found that older adults may face barriers to using web-based interventions, such as lack of computer skills and difficulties using the technology. For the development of future web-based interventions, it would appear necessary to consider the barriers that older adults face in using technology and find ways to overcome them. Including access to a coach through the web platform for initial and ongoing guidance could help reduce such barriers and, in turn, avoid discouragement among older adults committed to change [79]. In addition, the findings of this review indicate that older adults’ lifestyle habits are influenced by their perceptions of change in old age, as reported in other studies [73,75,79]. It would appear necessary to explore older adults’ perceptions of change and its benefits in future studies to promote change in this population.

In summary, we believe that the results of this review provide a better understanding of the components of web-based interventions that can lead to behavior change among older adults. In the studies identified, we found an overrepresentation of interventions focused on the PA behavior of older adults and conclude that other studies should be conducted to assess the effects on other lifestyle habits. The results of this review lead us to believe that authors should provide a more in-depth description of their interventions’ components, including the use parameters, BCTs, delivery modes, and theories used, to understand what is favorable to the adoption of a healthy lifestyle among other adults, how this is achieved, and how it could have influenced participants in behavior change. In particular, further studies should be carried out to understand how BCTs are used in an intervention, the impact of each of these BCTs, and the influence of the diverse delivery modes used on behavior change among older adults. Future web-based interventions should be based on one or more theories, and authors should indicate how these theories are used in the intervention to induce change. The results of this review suggest that further studies of web-based interventions to promote a healthy lifestyle in older adults should include support from a coach to develop a relationship of trust, seek to increase motivation among older adults, be tailored to older adults’ conditions, help them reduce barriers to using technology, and modify their perceptions of effecting change at their age. We propose that future web-based interventions be coconstructed with older adults to better identify their needs and what they seek, particularly with regard to support from a professional.

### Limitations

Although this is not the main objective or a necessary step in a scoping review, this review did not evaluate the quality of the studies, which may raise concerns about the rigor of the studies reviewed and affect the generalizability of the results. However, we critically reviewed all the studies. In addition, a language restriction (ie, only studies in English and French) was imposed, and this may have affected the exhaustiveness of the set of articles identified. In this review, we used a broad definition of older adults (ie, aged ≥65 years [2]). These results must be interpreted with caution given that older adults across the aging spectrum age differently and, regardless of their age, their needs may differ according to other characteristics such as comorbidities and frailty. Finally, step 6 of the Levac et al [28] framework (ie, consultation) was not completed as it was not relevant to the objectives of this scoping review. Indeed, this scoping review sought to explore the extent of the available literature on web-based interventions to promote healthy lifestyles among people aged ≥65 years, so consulting older adults would not have provided any insight into our subject. The consultation step may be more relevant in future studies conducted to map the needs of older adults in web-based interventions.

### Conclusions

This study identified components and outcomes of web-based interventions to promote healthy lifestyles among older adults. Although a variety of components were found, this scoping review revealed a positive trend in web-based interventions to promote healthy lifestyles, mostly through PA. More research is needed to further develop knowledge in this area, including examining the oldest old, evaluating the effects on various lifestyle habits such as diet and stress, clarifying how theories are integrated into the intervention, and discerning the contributions of each BCT and mode of delivery on the results obtained. Future web-based interventions among older adults should be coconstructed with them to ensure that the interventions are tailored to their conditions, limitations, and preferences; include the needed support of a coach; increase their motivation; help them modify their perceptions of behavior change; and reduce their barriers to using technology. Moreover, this study did not assess the quality of the literature, so the results must be interpreted with caution. With the current aging of the population, the growing use of the internet by older adults in recent years, and the pandemic context, which requires that we review how we provide care, it remains essential to continue developing and evaluating innovative, accessible interventions that will promote the health of older people while meeting the needs of an aging population. The results of this scoping review may inform health professionals and intervention developers about the relevant components and outcomes of web-based interventions in a population of older adults.

## Appendix

Multimedia Appendix 1 Search strategies.

Multimedia Appendix 2 PRISMA-ScR (Preferred Reporting Items for Systematic Reviews and Meta-Analyses extension for Scoping Reviews) checklist.

Multimedia Appendix 3 Characteristics of the studies.

Multimedia Appendix 4 Summary of the behavior change techniques used in the web-based interventions.

## Abbreviations

BCT: behavior change technique
CONSORT-EHEALTH: Consolidated Standards of Reporting Trials of Electronic and Mobile Health Applications and Online Telehealth
PA: physical activity
PRISMA: Preferred Reporting Items for Systematic Reviews and Meta-Analyses
PRISMA-ScR: Preferred Reporting Items for Systematic Reviews and Meta-Analyses extension for Scoping Reviews
