# Supplementary material for: Web-Based Interventions to Promote Healthy Lifestyles for Older Adults: Scoping Review
Source: Interact J Med Res. 2022 Aug 23;11(2):e37315. doi: 10.2196/37315 (PMC9449830; doi:10.2196/37315)
Supplement: Multimedia Appendix 1 [file ijmr_v11i2e37315_app1.docx]

# **Multimedia Appendix 1.** Search strategies

**MEDLINE**

((((((((((((Aged or Elder* or Senior? or Old) adj2 person) or Old) adj2 people) or Older) adj2 adult?) or Aging or Geriatric? or Gerontologic*) and (Web* or Internet? or Computer* or eHeath or Online*)).mp. and (Lifestyle? or "Life Style" or Lifestyle change? or Habit? or Behavioral change? or "Health behavio$r" or Physical activit? or Exercise? or Diet or Nutrition or Weight loss or Smoking or Smoking N2 Cessation or tabagism or Sedentary or "Sedentary behavior" or "Sedentary lifestyle" or Active Lifestyle? or Alcohol or "Alcohol drinking" or "Alcohol abuse" or Stress or "Stress management").ab,kf,kw,ti.) or (Aged and Internet).mp.) and (Life style.mp. or exp Exercise/ or Health behavior.mp. or Diet.mp. or Weight Loss.mp. or Stress, psychological.mp.)

**CINAHL**

(Aged OR Elder* OR Senior# OR Old* N2 person OR Old* N2 people OR Old* N2 adult# OR Aging OR Geriatric# OR Gerontologic*) AND (Web* OR Internet# OR Computer* OR eHeath OR Telemedicine OR Online* OR Telehealth OR telenursing) AND (Lifestyle# OR "Life Style" OR "Lifestyle change" OR Habit# OR Behavior* change# OR "Physical activit#" OR Exercise# OR Diet OR Nutrition OR "Weight loss" OR Smoking* OR Smoking N2 Cessation OR Tabagism OR Sedentary OR "Sedentary behavior" OR "Sedentary lifestyle" OR Active Lifestyle# OR Alcohol OR "Alcohol drinking" OR "Alcohol abuse" OR Stress OR "Stress management") OR ( (MH "Aged") OR (MH "Health Services for the Aged") OR (MH "Gerontologic Nursing+") OR (MM "In Old Age") ) AND (MH "Internet") AND ( (MH "Life style change") OR (MH "Health behavior") OR (MH "Life style, Sedentary") OR (MH "Habits+") OR (MH "Behavioral changes") OR (MH "Physical activity") OR (MH "Exercise+") OR (MH "Diet") OR (MH "Nutrition") OR (MH "Weight Loss") OR (MH "Stress") OR (MH "Alcohol drinking") )

**PsychInfo**

(Aged OR Elder* OR Senior OR Old* adj2 person OR Old* adj2 people OR Old* adj2 adult OR Aging OR Geriatric OR Gerontologic*) AND (Web* OR Internet OR Computer* OR eHeath OR Telemedicine OR Online* OR Telehealth OR telenursing) AND (Lifestyle OR "Life Style" OR "Lifestyle change" OR Habit OR Behavior*change OR "Physical activity" OR Exercise OR Diet OR Nutrition OR "Weight loss" OR Smoking* OR Smoking adj2 Cessation OR Tabagism OR Sedentary OR "Sedentary behavior" OR "Sedentary lifestyle" OR Active Lifestyle OR Alcohol OR "Alcohol drinking" OR "Alcohol abuse" OR Stress OR "Stress management")

**Web of Science**

(Aged OR Elder* OR Senior OR Old* NEAR/2 person OR Old* NEAR/2 people OR Old* NEAR/2 adult OR Aging OR Geriatric OR Gerontologic*) AND (Web* OR Internet OR Computer* OR ehealth OR Telemedicine OR Online* OR Telehealth OR telenursing) AND (Lifestyle OR "Life Style" OR "Lifestyle change" OR Habit OR Behavior*change OR "Physical activity" OR exercise OR Diet OR Nutrition OR "Weight loss" OR Smoking* OR Smoking NEAR/2 Cessation OR tabagisme OR Sedentary OR "Sedentary behavior" OR "Sedentary lifestyle" OR Active Lifestyle OR Alcohol OR "Alcohol drinking" OR "Alcohol abuse" OR Stress OR "Stress management") (Title) or (Aged OR Elder* OR Senior OR Old* NEAR/2 person OR Old* NEAR/2 people OR Old* NEAR/2 adult OR Aging OR Geriatric OR Gerontologic*) AND (Web* OR Internet OR Computer* OR ehealth OR Telemedicine OR Online* OR Telehealth OR telenursing) AND (Lifestyle OR "Life Style" OR "Lifestyle change" OR Habit OR Behavior*change OR "Physical activity" OR exercise OR Diet OR Nutrition OR "Weight loss" OR Smoking* OR Smoking NEAR/2 Cessation OR tabagisme OR Sedentary OR "Sedentary behavior" OR "Sedentary lifestyle" OR Active Lifestyle OR Alcohol OR "Alcohol drinking" OR "Alcohol abuse" OR Stress OR "Stress management") (Abstract) or (Aged OR Elder* OR Senior OR Old* NEAR/2 person OR Old* NEAR/2 people OR Old* NEAR/2 adult OR Aging OR Geriatric OR Gerontologic*) AND (Web* OR Internet OR Computer* OR ehealth OR Telemedicine OR Online* OR Telehealth OR telenursing) AND (Lifestyle OR "Life Style" OR "Lifestyle change" OR Habit OR Behavior*change OR "Physical activity" OR exercise OR Diet OR Nutrition OR "Weight loss" OR Smoking* OR Smoking NEAR/2 Cessation OR tabagisme OR Sedentary OR "Sedentary behavior" OR "Sedentary lifestyle" OR Active Lifestyle OR Alcohol OR "Alcohol drinking" OR "Alcohol abuse" OR Stress OR "Stress management") (Author Keywords)

**Cochrane Database of Systematic Reviews**

(Aged OR Elder* OR Senior OR Old* adj2 person OR Old* adj2 people OR Old* adj2 adult OR Aging OR Geriatric OR Gerontologic*) AND (Web* OR Internet OR Computer* OR eHeath OR Telemedicine OR Online* OR Telehealth OR telenursing) AND (Lifestyle OR "Life Style" OR "Lifestyle change" OR Habit OR Behavior*change OR "Physical activity" OR Exercise OR Diet OR Nutrition OR "Weight loss" OR Smoking* OR Smoking adj2 Cessation OR Tabagism OR Sedentary OR "Sedentary behavior" OR "Sedentary lifestyle" OR Active Lifestyle OR Alcohol OR "Alcohol drinking" OR "Alcohol abuse" OR Stress OR "Stress management")

**Joanna Briggs Library**

((((((((((Aged or Elder* or Senior or Old*) adj2 person) or Old*) adj2 people) or Old*) adj2 adult) or Aging or Geriatric or Gerontologic*) and (Web* or Internet or Computer* or eHeath or Telemedicine or Online* or Telehealth or Telenursing) and (((Lifestyle or "Life Style" or "Lifestyle change" or Habit or Behavior*change or "Physical activity" or Exercise or Diet or Nutrition or "Weight loss" or Smoking* or Smoking) adj2 Cessation) or Tabagism or Sedentary or "Sedentary behavior" or "Sedentary lifestyle" or Active Lifestyle or Alcohol or "Alcohol drinking" or "Alcohol abuse" or Stress or "Stress management")).ab. or ((((((((((Aged or Elder* or Senior or Old*) adj2 person) or Old*) adj2 people) or Old*) adj2 adult) or Aging or Geriatric or Gerontologic*) and (Web* or Internet or Computer* or eHeath or Telemedicine or Online* or Telehealth or Telenursing) and (((Lifestyle or "Life Style" or "Lifestyle change" or Habit or Behavior*change or "Physical activity" or Exercise or Diet or Nutrition or "Weight loss" or Smoking* or Smoking) adj2 Cessation) or Tabagism or Sedentary or "Sedentary behavior" or "Sedentary lifestyle" or Active Lifestyle or Alcohol or "Alcohol drinking" or "Alcohol abuse" or Stress or "Stress management")).kw. or ((((((((((Aged or Elder* or Senior or Old*) adj2 person) or Old*) adj2 people) or Old*) adj2 adult) or Aging or Geriatric or Gerontologic*) and (Web* or Internet or Computer* or eHeath or Telemedicine or Online* or Telehealth or telenursing) and (((Lifestyle or "Life Style" or "Lifestyle change" or Habit or Behavior*change or "Physical activity" or Exercise or Diet or Nutrition or "Weight loss" or Smoking* or Smoking) adj2 Cessation) or Tabagism or Sedentary or "Sedentary behavior" or "Sedentary lifestyle" or Active Lifestyle or Alcohol or "Alcohol drinking" or "Alcohol abuse" or Stress or "Stress management")).ti.

**Open Grey**

(Aged OR Elderly OR Senior OR Older person OR Older people OR Older N2 adult OR Aging OR Geriatric OR Gerontologic) AND (Web OR Website OR Internet OR Computer OR eHeath OR Telemedicine OR Online OR Telehealth OR telenursing) AND (Lifestyle OR "Life Style" OR "Lifestyle change" OR Habit OR Behavioral change OR "Physical activity" OR Exercise OR Diet OR Nutrition OR "Weight loss" OR Smoking OR Smoking Cessation OR Tabagism OR Sedentary OR "Sedentary behavior" OR "Sedentary lifestyle" OR Active Lifestyle OR Alcohol OR "Alcohol drinking" OR "Alcohol abuse" OR Stress OR "Stress management")

**Google Scholar**

allintitle: web-based intervention elderly

allintitle: web-based intervention older adults

allintitle: ehealth lifestyle older adults

allintitle: ehealth lifestyle elderly

allintitle: ehealth behavior change older adults

allintitle: ehealth behavior change elderly

allintitle: web behavior change older adults

allintitle: web behavior change elderly

allintitle: web physical activity older adults

allintitle: web physical activity elderly

allintitle: web stress older adults

allintitle: web stress elderly

allintitle: web diet older adults

allintitle: web diet elderly

allintitle: web smoking elderly

allintitle: web smoking older adults

allintitle: web weight loss elderly

allintitle: web weight loss older adults

allintitle: web alcohol loss elderly

allintitle: web alcohol older adults
