# Supplementary material for: Web-Based Interventions to Promote Healthy Lifestyles for Older Adults: Scoping Review
Source: Interact J Med Res. 2022 Aug 23;11(2):e37315. doi: 10.2196/37315 (PMC9449830; doi:10.2196/37315)
Supplement: Multimedia Appendix 3 [file ijmr_v11i2e37315_app3.docx]

**Multimedia Appendix 3.** Characteristics of the studies

| **Authors, country of publication** | **Aim** | **Design and sample** | **Intervention** | **Principal results** |
| --- | --- | --- | --- | --- |
| Alley et al. (2019) [43]  Australia | To determine the changes in objectively measured MVPA of participants assigned to a web-based computer-tailored physical activity program for older adults (with and without activity tracker integration) compared with a control group. | **Design**: Research protocol of a randomized controlled trial  **Sample**: 300 older adults aged 65 and over who do not meet the recommendations for recommended physical activity (30 minutes 5 times/week) | IG 1: Active for life  IG 2: Active for life + activity tracker (i.e., FitBit)  CG: waiting list | N/A |
| Volders et al. (2019) [44]  Netherlands | To describe the protocol of a clustered randomized controlled intervention trial that investigate the short-term (6 months) and long-term (12 months) effects of the Active Plus program on CF of people aged 65years or older with chronic illness(es). | **Design:** Research protocols of a two-group cluster randomized controlled trial  **Sample:** 540 older adults aged 65 and over (x=74,5 years old) who suffer from at least one chronic disease that affect or can affect mobility and that are able to walk 100m without help | IG: Active Plus  CG: Waiting list | N/A |
| Volders et al. (2020) [45]  Netherlands | To describe the effects of the Active Plus intervention on PA of older adults with chronic illnesses, compared to a waiting list control group | **Design**: Two-group cluster randomized controlled trial  **Sample**: 585 older adults aged 65 and over (x=74,5 years old) who suffer from at least one chronic disease that affect or can affect mobility and that are able to walk 100m without help | IG: Active Plus  CG: Waiting list | **At six months:** No difference between groups for objective measure of physical activity.  Increasing likelihood to perform self-reported cycling (p=0,01) and gardening (p=0,04) in the IG.  **At 12 months:** No difference between groups for objective measure of physical activity.  Increasing likelihood to perform self-reported walking in the IG (p=0,08). |
| Boekhout et al. (2018) [46]  Netherlands | To provide insight into the effects of the Active Plus65 intervention on PA and it examines whether the adaptations made to Active Plus65 result in comparable or even better results than the original proven effective intervention that was less adapted to this specific target population. | **Design:** Quasi-experimental pre-test post-test  **Sample:** 502 older adults aged 65 and over (x=76,75 in the IG and x=74,36 years old in the CG) with an impairment in physical activity caused by a non-communicable chronic disease | IG: Active Plus 65  CG: Active Plus 50, a web-based intervention intended for older adults aged 50 and over | **At 3 months:** Increasing weekly minutes of moderate to vigorous physical activity (p<0,001) and in the days per week with sufficient MVPA (p<0,001) in the IG.  No significant differences between IG and CG for the days per week with sufficient MVPA between T0, T1, and T2.  **At 6 months:** Decreasing days per week with sufficient MVPA but still significant between t0 and 6 months (p=0,001) in IG.  Between groups, a significant difference for weekly minutes of MVPA was found in favor of the CG (p=0,004). |
| Boekhout et al. (2019) [47]  Netherlands | To determine (1) which individual characteristics predict differences in delivery mode preference between the printed or Web-based delivery mode and (2) which user characteristics and delivery mode predict attrition. | **Design**: Pre-test post-test study  **Sample**: 409 older adults aged 65 and over with a chronic disease (x=79,22 in the CG and x=73,29 years old in the IG) | IG: Active Plus 65  CG: Active Plus 65 in a printed format | 41% selected Web version of the intervention.  Web-based participants were younger (p<0,001) and had a higher level of social support (p=0,001).  Attrition rate was 71% in web format versus 50% in printed format. |
| Pothier et al. (2018) [52]  France | To describe the protocol of the eMIND study, a randomized controlled trial using a web-based multi- domain intervention for older adults. | **Design**: Research protocol of a pilot randomized controlled trial  **Sample**: 120 community-dwelling older adults aged 65 and over (x= 74,2 years old) who present a subjective memory complaint, without dementia. | IG: eMind  CG: Website with information on aging and health. Monthly phone call from the research team. | N/A |
| De Souto Barreto et al. (2021) [51]  France | To describe the feasibility and acceptability of a 6-month randomized controlled trial (RCT) of a web- based multidomain lifestyle training intervention for community-dwelling older people with spontaneous memory complaint and to test the effects of the intervention on both function- (e.g., cognition, mobility) and lifestyle-related (e.g., PA, food intake) outcomes. | **Design**: Pilot parallel-group randomized controlled trial  **Sample**: 120 community-dwelling older adults aged 65 and over (x= 74,2 years old) who present a subjective memory complaint, without dementia. | IG: eMind  CG: Website with information on aging and health. Monthly phone call from the research team. | No significant change on cognitive function, nutrition, or physical activity. More contact with health professionals would have been helping. Motivating rewarding system could have increase interest and adherence.  63,5% of participants adhered to the cognitive training, 60,3% to nutrition and 5,2% to exercise training.  Exercises were too easy, repetitive, difficult to perform due to space limitations at home, and progressed slowly, nutritional advice was lacking novelty, cognitive training was repetitive and too long. |
| Garcia-Camacha et al. (2020) [38]  Spain | To evaluate the impact of the Healthy Ageing Supported by Internet and Community training program for acquiring the knowledge and skills necessary for a healthy lifestyle in community- dwelling adults over 65 years of age | **Design**: Pre-test post-test pilot study  **Sample**: 24 older adults aged 65 and over (x=69,45 years old) | IG: Healthy Ageing Supported by Internet and Community  CG: N/A | Increase in knowledge and skills to adopt a healthy lifestyle (p<0,05 for all items). |
| Jongstra et al. (2017) [50]    Netherlands | To describe the full development of an interactive Inter- net platform for older people, which was designed for the Healthy Ageing Through Internet Counselling in the Elderly (HATICE) study. | **Design:** Pilot randomized controlled trial  **Sample:** 49 older adults aged 65 and over (x=69 years old) at high risk of cardiovascular disease | IG: Healthy Ageing Through Internet Counseling in the Elderly (HATICE)  CG: static website with information on cardiovascular risk factor | The majority of the goals fixed by the participants were related to improvement of exercises and weight loss.  Participants considered an instruction video necessary. Setting a goal was difficult for the participants.  Healthy lifestyle information were appreciated, but the participants preferred to print the texts.  Participants appreciated interactives components (setting goals, videos, electronic diary) and the discussions with the coach. Communication felt personal. |
| Barbera et al. (2018) [48]  Finland | To design a multidomain intervention to improve CVR, based on the guidelines for CVR management, and administered through a coach-supported, interactive, platform to over 2500 community-dwellers aged 65+ in three European countries. | **Design:** Qualitative (comparative analysis of national and European guideline for primary and secondary cardiovascular disease prevention)  **Sample:** N/A | IG: HATICE  CG: N/A | Lifestyle-related recommendations were consistent between country. Key differences were methods used to assess the cardiovascular risk factor (e.g., sum of the cardiovascular risk factor in the French guideline versus calculated risk of developing a cardiovascular disease within 5 or 10 years in the other country), but it did not hamper the intervention design. Minor country-specific adaptations were implemented to maximize the intervention feasibility (e.g., nutrition recommendation adapted to local food habits). |
| Coley et al. (2019) [19]  France | To explore older adults’ reasons for participating in a European multinational eHealth prevention trial, and to compare motivations between countries. | **Design:** Cross-sectional mixed methods research  **Sample:** 341 older adults aged 65 and over (x=68,7) at high risk of cardiovascular disease | IG: HATICE  CG: N/A | Main reason for participating was personal benefits (some participants hoped that the intervention could offer them external support and motivation and benefit from additional medical monitoring).  Participants appreciated to have access to a continuous intervention.  Barriers were lack of confidence in computer skills. |
| Richard et al. (2016) [49]  Netherlands | To investigate whether a coach-supported interactive internet intervention to optimize self-management of cardiovascular risk factors in older individuals can improve the cardiovascular risk profile and reduce the risk of cardiovascular disease and cognitive decline. | **Design**: Research protocol of a prospective open-label blinded endpoint clinical trial  **Sample**: 2600 older adults aged 65 and over at high risk of cardiovascular disease | IG: HATICE  CG: Static website with information on cardiovascular risk factor | N/A |
| Richard et al. (2019) [12]  Netherlands | To investigated whether a coach-supported interactive internet intervention to optimize self-management of cardiovascular risk factors in older adults can improve cardiovascular risk profiles and reduce the risk of cardiovascular disease and dementia | **Design**: Prospective open-label blinded endpoint clinical trial  **Sample**: 2624 older adults aged 65 and over (x=69 years old) at high risk of cardiovascular disease | IG: HATICE  CG: Static website with information on cardiovascular risk factor | Improved systolic blood pressure, lipid, and body mass index (p=0,008) in favor of the intervention group.  Smoking cessation was reported by 23,5% of smokers in the GI versus 14,2% in GC (mean difference 9,4%; 95% CI -1,1 to 19,8).  Number of logins was 59441 in the IG versus 17014 in the CG. |
| van Middelaar et al. (2018) [20]  Netherlands | to study older peoples’ experiences with an interactive internet platform for cardiovascular self-management, to assess which factors influence initial and sustained engagement | **Design**: Qualitative  **Sample**: 20 older adults aged 65 and over at high risk of cardiovascular disease (n=n/d) | IG: HATICE | Developing a relationship with the coach was needed for participants to engage in change.  Some of the participants find the message system insufficient to develop a relationship with the coach. A first in person meeting with the coach would have been appreciated.  Participants that felt connected to the coach or perceived positive results on their health felt inclined to keep using the platform and adhere to their goals. Difficulty of use or no internet skills discouraged older adults. |
| Van Bruissen et al. (2017) [42]  Netherlands | To outline how elderly and their health professionals were involved in the development and implementation of the platform and how their input influenced the design, functionality, and implementation of the platform. | **Design**: Descriptive  **Sample**: 16 prefrail older adults (x=83 years old) | IG: Life project  GC: N/A | Participants preferred tailored advice to their limitation and preference.  Being physically active was not a goal in itself for the participants. Social contacts and staying independently as long as possible motivated them.  Problems with the intervention were: 1) safety and insecurity (e.g.. fear of getting injured) 2) usability issues (e.g., old software) and 3) lack of discipline to exercise alone at home. |
| Dyck et al. (2019) [41]  Belgium | To investigate the effect of the theory-based eHealth intervention, MyPlan 2.0, focusing on pre- and post-intentional determinants on both accelerometer-based and self-reported PA levels in older Belgian adults in the short and intermediate term. | **Design**: Randomized controlled trial  **Sample**: 72 older adults aged 65 to 80 years old (x=70,9 years old), able to walk 100m without help | IG: MyPlan 2.0  CG: No intervention | **Post-intervention:** Increased self-reported moderate leisure time physical activity (p=0,09) in favor of CG. Increased total of objectively measured physical activity (p=0,07).  **At 3 months:** Increased MVPA (p=0,07) in favor of IG. Increased self-reported vigorous leisure time physical activity (p=0,02) in favor of IG. No significant intervention effects for overall leisure-time PA. |
| Baez et al. (2016) [39]  Italy | To describe the design and validation of a virtual fitness environment aiming at keeping older adults physically and socially active. | **Design**: Qualitative  **Sample**: 40 not frail community-dwelling older adults aged 65 and over (x= 71 years old) | IG: Otago  CG: Same intervention without social component. | Persuasive features (monitor progress) and messaging the coach were the most appreciated components.  Less used components were messaging other members appreciated factor was Internet problem. |
| Baez et al. (2017) [40]  Italy | 1) To investigate if and how online group-exercising and baseline measures of physical, social and psychological wellbeing influence the adherence of older adults to the training program.  2) To assess the effectiveness of an OTAGO-based exercise program delivered via an online group-exercising tool - effectiveness measured as the improvements in the physical functions expected by the exercise program. | **Design**: Matched randomized trial  **Sample**: 40 community-dwelling older adults aged 65 and over (x= 71 years old) who are not frail | IG: Otago  CG: Same intervention without social component. | No effect was found between group and time for leg muscle strength, gait speed and enjoyment of physical activity.  The number of messages received was correlated with the improvement in the loneliness scores (p=0,003).  Increased well-being in the two groups (p=0,034). |
| Bickmore et al. (2013) [37]  United-States | To test the efficacy of an embodied conversational agent-based physical activity intervention designed for older adults that could be deployed from outpatient clinics, targeting individuals who are in need of greater mobility. | **Design**: Randomized controlled trial  **Sample**: 263 inactive older adults aged 65 and over (x=71,3 years old) who didn’t perform 20 min of MVPA 3 times/week during the last six months | IG: No name  CG: No intervention, pedometers only | Increased number of daily steps at 2 months (p=0,01) in favor of IG, no longer significant at 12 months (p=0,09).  80% of the participants affirmed that the coach helped them to walk more. |
| Pettersson et al. (2019) [36]  Switzerland | To explore older people’s experiences of a self-management fall prevention exercise routine guided either by a digital program or a booklet. | Design: Qualitative  Sample: 28 older adults aged 70 and over (x=76 years old) with self-reported impaired balance, able to rise from a highchair and stand without support, and who are not active (doing intense exercise more than 3 hours/week) | IG: No name  CG: Paper booklet of exercise | Choosing exercises was for the most part perceived as easy and appreciated, except for those with pain or medical problem. This could set the limits for what exercises they felt were possible to choose.  The flexibility to be able to do the exercise in their own time and place was for the most part found to be positive.  Participants in both groups described that discipline was required to follow their routines.  Identifying their own driving force for doing the exercises (health, improvements, encouragement of relatives) was important to maintain their routines. |

IG: Intervention group; CG: Control group; N/A: MVPA: Moderate to vigorous physical activity; N/A: Not applicable; PA: Physical activity
