# Supplementary material for: Web-Based Interventions to Promote Healthy Lifestyles for Older Adults: Scoping Review
Source: Interact J Med Res. 2022 Aug 23;11(2):e37315. doi: 10.2196/37315 (PMC9449830; doi:10.2196/37315)
Supplement: Multimedia Appendix 4 [file ijmr_v11i2e37315_app4.docx]

**Multimedia Appendix 4.** Summary of behavior change techniques used in the web-based interventions

|  | Active for life [43] | Active Plus  [44, 45] | Active Plus 65  [46, 47] | eMind  [51, 52] | HASIC  [38] | HATICE  [12, 19, 20, 48-50] | Life Project [42] | MyPlan 2.0 [41] | Otago [39, 40] | No name [37] | No name [36] | Total (n=) |
| --- | --- | --- | --- | --- | --- | --- | --- | --- | --- | --- | --- | --- |
| Instruction | X | X | X | X | X | X | X | X | X | X | X | 11 |
| Goal setting | X |  |  |  |  | X |  | X |  | X |  | 4 |
| Self-monitoring | X |  |  | X | X | X |  | X | X | X | X | 8 |
| Action planning | X | X |  |  |  |  |  | X |  |  | X | 4 |
| Feedback | X | X | X | X |  | X |  | X | X | X | X | 9 |
| Problem solving |  |  | X |  |  | X |  | X |  | X |  | 4 |
| Verbal persuasion |  |  |  |  |  |  |  |  | X |  |  | 1 |
| Commitment |  | X |  |  |  |  |  |  |  |  |  | 1 |
| Awareness |  |  | X |  |  |  |  |  |  |  |  | 1 |
| Coping planning |  | X | X |  |  |  |  |  |  |  |  | 2 |
| Self-regulation |  | X |  |  |  |  |  |  |  |  |  | 1 |
| Prompt and cues | X |  |  |  |  |  |  |  |  |  |  | 1 |
| Rewards | X |  |  |  |  |  |  |  |  |  |  | 1 |
| Relapse prevention | X |  |  |  |  |  |  |  |  |  |  | 1 |
| Social comparison | X |  |  |  |  |  |  |  |  |  |  | 1 |
| Total (n=) | 9 | 6 | 5 | 3 | 2 | 5 | 1 | 6 | 4 | 5 | 4 |  |
